# A Meta-Analysis on Remote HRI and In-Person HRI: What Is a Socially Assistive Robot to Do?

**DOI:** 10.3390/s22197155

**Published:** 2022-09-21

**Authors:** Nan Liang, Goldie Nejat

**Affiliations:** 1 Autonomous Systems and Biomechatronics Laboratory (ASBLab), Department of Mechanical and Industrial Engineering, University of Toronto, 5 King’s College Rd, Toronto, ON M5S 3G8, Canada; 2 KITE, Toronto Rehabilitation Institute, Toronto, ON M5G 2A2, Canada; 3 Rotman Research Institute, Baycrest Health Sciences, Toronto, ON M6A 2E1, Canada

**Keywords:** remote and in-person human–robot interaction, socially assistive robots, robot embodiment, robot presence, user experience and perceptions

## Abstract

Recently, due to the COVID-19 pandemic and the related social distancing measures, in-person activities have been significantly reduced to limit the spread of the virus, especially in healthcare settings. This has led to loneliness and social isolation for our most vulnerable populations. Socially assistive robots can play a crucial role in minimizing these negative affects. Namely, socially assistive robots can provide assistance with activities of daily living, and through cognitive and physical stimulation. The ongoing pandemic has also accelerated the exploration of remote presence ranging from workplaces to home and healthcare environments. Human–robot interaction (HRI) researchers have also explored the use of remote HRI to provide cognitive assistance in healthcare settings. Existing in-person and remote comparison studies have investigated the feasibility of these types of HRI on individual scenarios and tasks. However, no consensus on the specific differences between in-person HRI and remote HRI has been determined. Furthermore, to date, the exact outcomes for in-person HRI versus remote HRI both with a physical socially assistive robot have not been extensively compared and their influence on physical embodiment in remote conditions has not been addressed. In this paper, we investigate and compare in-person HRI versus remote HRI for robots that assist people with activities of daily living and cognitive interventions. We present the first comprehensive investigation and meta-analysis of these two types of robotic presence to determine how they influence HRI outcomes and impact user tasks. In particular, we address research questions regarding experience, perceptions and attitudes, and the efficacy of both humanoid and non-humanoid socially assistive robots with different populations and interaction modes. The use of remote HRI to provide assistance with daily activities and interventions is a promising emerging field for healthcare applications.

## 1. Introduction

With robots becoming more common in people’s everyday lives, the field of human–robot interaction (HRI) has been rapidly expanding [1,2,3]. In particular, socially assistive robots (SARs) have been developed to help address many societal challenges such as an aging population and the increased demand for healthcare [4,5,6]. Namely, SARs have been developed to aid with activities of daily living (ADLs) including meal preparation and eating [7,8,9], clothing recommendation and dressing [10], monitoring [11,12,13], reminders [14,15,16], rehabilitation [17,18,19], and social behavioral interventions for children living with autism [20,21,22]. 

Due to the social distancing measures introduced during the COVID-19 pandemic, in-person activities have been significantly reduced to limit the spread of the virus, especially in healthcare settings [23]. This has led to the development of several new HRI scenarios for SARs including remote education and tutoring [24], remote presence through robots at job fairs [25], and robot-based video interventions for social and cognitive development [26,27]. However, loneliness and social isolation are a concerning result of the pandemic especially for our most vulnerable populations [28]. Therefore, SARs can be used to play a vital role in reducing the negative affects of social isolation on physical, emotional, and cognitive health [23], not just during the pandemic but also in a post-pandemic society for such populations. SARs have the ability to provide social and cognitive assistance with both the activities of daily living including self-care and hygiene, and with cognitively and physically stimulating activities such as memory and logic games, and exercise [8]. The interactions that SARs provide can be tailored to different populations ranging from children with developmental needs to older adults living with dementia. The ongoing COVID-19 pandemic has accelerated the exploration of remote interactions in workplaces scenarios through telework and virtual meetings to homes and healthcare settings through remote patient monitoring and telehealth [29]. Recently, the use of remote HRI by social robots in providing cognitive assistance directly at home has also been explored [26,27].

**Figure 1:**
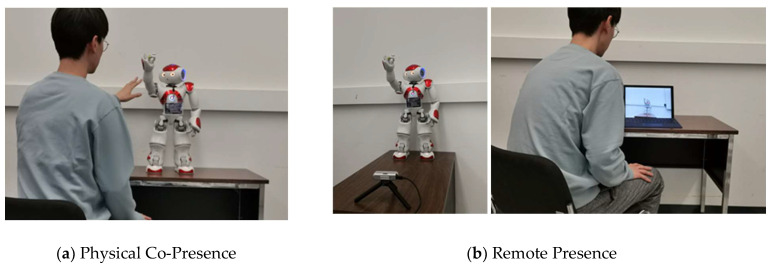
(**a**) In-person HRI scenario, where robot and user are in the same location; and (**b**) Remote HRI scenario, where robot and user are spatially separated in different locations.

In general, social HRI can be facilitated with two main types of physical robot presence [30,31]: (1) in-person HRI: where interaction is with a co-present or collocated robot, and the robot and users are located in the same physical space, or (2) remote HRI where the robot and users are not collocated and are spatially separated. In-person HRI allows for interaction with physically embodied robots via physical co-presence, whereas remote HRI considers interactions with a physically embodied robot via remote presence, as shown in Figure 1. 

The first study comparing in-person and remote HRI was conducted in 2004 where participants responded to requests from a humanoid robot for a dessert-serving task and a teaching task, and no significant difference was found between in-person and remote HRI [32]. More recent studies have shown that, through both types of HRI, people can successfully achieve similar performances [33,34] and have comparable perceptions towards these robots [35,36]. Remote HRI can provide several benefits to in-person HRI: (1) it can minimize the presence of other individuals whether they are experimenters or care staff that need to set up the robots for interactions, and (2) it also allows for the potential scaling-up of robot use as the same robot can be remotely used by different people and across diverse settings from private homes to long-term care homes. Compared to remote human–human interaction (HHI), the use of remote HRI can potentially help to alleviate staff shortages [37,38,39] and high healthcare costs [40,41,42], as well as caregiver burnout and workload [43,44,45] by providing needed interventions [26,27], monitoring [46], and disease management [47], especially during the COVID-19 pandemic. Existing in-person and remote comparison studies have investigated the feasibility of these types of HRI on individual scenarios and tasks, e.g., [33,34,35,36,46,48,49,50,51,52,53,54,55]. However, no consensus on the specific differences between in-person HRI and remote HRI have been determined, as contradictory results have been reported. In [30], a 2015 survey reviewed physical embodiment and physical presence in 33 different studies using simple counting and concluded that in-person HRI promotes more positive responses from users than remote HRI (with a physical or virtual robot). However, the exact outcomes for in-person HRI with a physical socially assistive robot versus remote HRI with a physical socially assistive robot have not been extensively compared and their influence on physical embodiment in remote conditions has not been addressed or quantitively analyzed to date. Therefore, the direct impact of the role of “robot presence” is still not known. In this paper, we investigate and compare in-person HRI versus remote HRI for robots that assist people with the activities of daily living and cognitive interventions. We present the first comprehensive investigation and meta-analysis of these two types of robotic presence to determine how they influence HRI outcomes and impact user tasks. In particular, we address research questions regarding experience, perceptions and attitudes, and efficacy of both humanoid and non-humanoid SARs with different populations and tasks.

## 2. Related Works

In this section, we review separate studies on in-person HRI and remote HRI assistance to identify and motivate outcomes and advantages of both types of HRI scenarios. 

### 2.1. In-Person Robot Assistance

There have been numerous in-person HRI studies throughout the past few decades showing the potential for robot assistance for both physical tasks [56] and cognitive tasks [57,58,59,60], with the aim of enhancing mobility and functionality [56], improving disease management [57], reducing staff workload [58], and providing needed interventions [8,60]. 

With respect to cognitive tasks, in [57], an 8-week trial with a social robot was conducted in a hospital to help with Type-1 diabetes management. A NAO robot was used to deliver two in-person sessions and two pre-recorded sessions (displayed on a television) of behavioral interventions with mental imagery to the patients in order to reduce unhealthy drink and food consumption. The program was found to help two out of 10 participants reduce their unhealthy diets by 70%.

In [58], a Pepper robot was used to lead physical exercise and social activities (e.g., singing songs) for older adults with dementia in a hospital setting. The activities were facilitated by the robot with no supervision from the healthcare professionals. A post-study survey found that 25% of the participating healthcare professionals found the robot decreased their workloads.

In [8], the expressive socially assistive robot Brian 2.1 was developed for providing assistance to the older adults including those living with cognitive impairments. A study in a long-term care facility showed that the robot was able to assist with meal-eating and memory card games, and users had positive attitudes towards the robot and its assistive behaviors and found the robot easy to use. 

In [60,61], an interactive robot Tangy was developed to autonomously facilitate cognitive and socially stimulating games with older adults. HRI studies were conducted at long-term care centers with Tangy facilitating both Bingo and team-based Trivia games. Participants had high engagement and compliance for both games and had an overall positive experience with the robot. Furthermore, the robot promoted social interactions between the participants.

### 2.2. Remote Robot Assistance

To date, there has been only a handful of remote HRI studies [26,27,47] with social robots. For example, in [47], the NAO robot was used to interact remotely through tele-conferencing with diabetic children and encourage them to keep a diary. By comparing the diary entries of six participants before and after the robot interactions, it was found that children with support from the remote NAO wrote more in their diaries than those without robot support. They also shared significantly more about their personal experiences in their diaries when interacting with NAO.

In [26], the feasibility of using remote HRI for delivering special education (communication skills, dance and breathing exercises) to children living with Autism Spectrum Disorder (ASD) was explored. As an alternative to in-person treatments, video educational presentations with (robot-assisted group) and without (control group) the NAO robot were delivered in an asynchronous manner to children with ASD; and then live synchronous therapy sessions with NAO were conducted. Compared to the control group, the robot-assisted group showed higher ratings for satisfaction, engagement, and perceived usefulness on a Likert-scale questionnaire. A similar exploratory study with three children with ASD during the COVID-19 pandemic was presented in [27] to explore the effects of using remote HRI for ASD intervention. The NAO robot provided assistance to users on how to initiate and continue a conversation, and the robot also danced with the children. The authors concluded that remote HRI was able to successfully stimulate interaction capabilities based on verbal, facial and body expressions of the participants. There has been experimental evidence showing that people can have emotional responses, including empathy, towards non-collocated robots [62].

### 2.3. Summary

To date, in-person and remote social HRI have been successfully used to provide support and assistance to different groups, mainly for disease management [47,57], cognitive interventions [26,27,58], and assisting with the activities of daily living [8,27,58]. They both have had positive outcomes on users. Namely, in-person HRI has been found to be effective for interventions [57], has the potential to reduce staff workload [58], and robots in such scenarios have been found to be easy to use [8] and engaging [60,61]. Remote HRI has been shown to be stimulating [27], engaging and useful [26], and users have also expressed trust and closeness to these remote robots [47]. As similar assistive tasks can be achieved by both types of HRI, it is important to investigate and compare if users specifically perceive in-person and remote HRI differently and how this influences their overall experience for various assistive activities and scenarios. 

## 3. Methodology

The objective of this study is to conduct a quantitative meta-analysis between in-person HRI and remote HRI for socially assistive robots. We use a meta-analysis approach to statistically combine and consolidate the results (which may be conflicting) of various independent in-person versus remote HRI studies to generate a reliable and accurate overall estimate of their effects and outcomes. The criteria and procedures we utilize are explained in detail below. 

### 3.1. Meta-Analysis Criteria

The first step in our literature review process was to conduct a systematic search to identify HRI comparison studies between robot in-person and remote conditions. The inclusion criteria we used was: (1) HRI should be between a physical embodied robot for both in-person and remote conditions, (2) the robot should be assisting a user(s) with health- or wellbeing-related activities, and (3) quantitative results and/or descriptive statistics are reported. 

**Figure 2:**
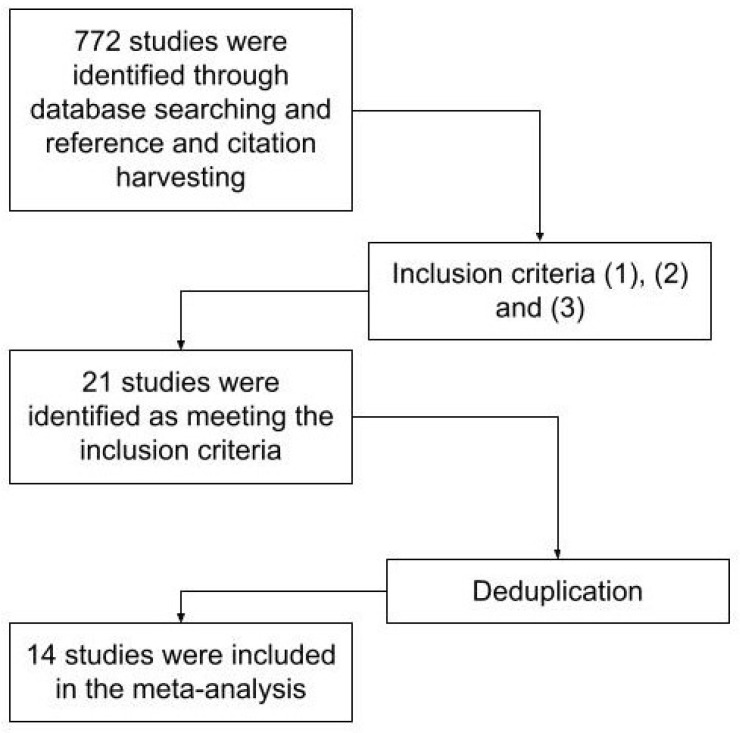
Flowchart of study selection procedure.

A meta-search was first conducted using databases including IEEE Xplore, Scopus, PubMed, SAGE Journals, PsychINFO, SpringerLink, ScienceDirect, ACM Digital and Google Scholar. Keywords used to search the databases included “robot”, “remote”, “in-person”, “HRI”, “embodiment” and “presence”. Our second step included reference harvesting and citation harvesting. A total of 772 papers were found and examined, and 21 studies were further considered based on our criteria. Taking into account duplications, 14 unique HRI studies were included in this meta-analysis using our procedure as shown in Figure 2. These studies are summarized in Table 1 and discussed below.

#### 3.1.1. Studies with Differences between Outcomes for in-Person and Remote HRI

In [49], an ActivMedia Pioneer 2 DX mobile robot was used for coaching the cognitive game Towers of Hanoi puzzle in remote and in-person conditions. As for the remote conditions, the robot was displayed on a screen in front of the user over real-time video-conferencing. Thirty-two adults with an average age of 24.7 years participated in this study. Game performance (e.g., total game time, optimal moves) was measured, and a questionnaire was used to rate the different conditions. Task performance was higher for the in-person condition over the remote condition, and participants found the in-person condition more helpful and enjoyable. 

In [54], the upper-torso robot Nico was used to prompt adult users to complete certain tasks in a home-like environment. A total of 22 participants were recruited from a university for the in-person condition and 22 participants for the remote condition. In both conditions, greetings, cooperation, trust, and personal space were measured based on task completion rates, task reaction times and distance to the robot. A Likert-scale questionnaire was used to measure perceptions towards the robot. The results showed that participants in the in-person conditions had higher task success rates and lower reaction times, especially when fulfilling the unusual task of throwing books into garbage bins. Participants also found the in-person HRI to be more natural than the remote HRI.

In [50], the chick-like Keepon robot was used to help undergraduate and graduate students complete nonogram puzzles. One hundred participants were asked to solve the puzzles on a laptop with the robot providing advice on player moves. In the in-person conditions, the physical robot was placed next to the laptop, and in the remote conditions, it was displayed together with a puzzle on the screen. Task performance was measured based on solution time. A Likert-scale questionnaire was used to measure relevance, understandability, and distraction of the robot. It was found that participants achieved higher task performance with the in-person HRI. A statistically significant difference between the two conditions was found for robot distraction with higher values for the remote condition; however, no significance was found with respect to robot understandability and relevance.

In [52], Robovie R3 was used to tutor children in sign language. In total, 31 children with hearing impairments were asked to recognize the sign performed by the in-person robot and the remote robot displayed on a screen. Task performance was measured by sign language recognition accuracy. It was found that the participants were able to recognize the sign language symbols with higher accuracy in the in-person HRI condition versus the remote HRI scenario. 

In [55], NAO was used to help adults find the correct corresponding relationships in figures consisting of different shapes. In total, 60 undergraduate and graduate students participated in the experiment, answering 10 questions by verbally selecting the correct option displayed on a screen for a given question. Based on their initial answers, verbal feedback was provided by the robot and then participants decided whether they would follow the robot’s feedback. The decision changing rate was measured. A Likert questionnaire was also used to measure participants’ faith, attachment, social presence, and credibility towards the robot. The in-person interaction was found to have more influence on participants’ decision-making for the questions, and also was favored over remote HRI in terms of faith, attachment, and credibility.

In [46], RoboThespian was used for prompting users to follow a set of verbal instructions in a shopping mall, including greetings, engaging in casual talks and requesting to take photos of the participants. In the remote condition, the robot was displayed using an LED screen in the mall. The task completion rates were designed to measure the proactivity, reactivity, commitment, and compliance levels. In total, 7685 participants (mostly adults) participated in the study. Results showed that in-person interactions with RoboThespian had higher proactivity, reactivity, commitment; however they did not have compliance. 

In [48], conversations related to health habits took place between the nurse robot Pearl and adult participants. A set of questions were asked by Pearl and replies from the participants were collected through keyboard entries. In the remote condition, the robot was projected on a screen. Measurements included both objective measures of conversion (e.g., time with the robot) and self-reported measures from a Likert-scale questionnaire on attitudes towards the robot. The results showed the in-person HRI conditions were more engaging, influential, and anthropomorphized. 

In [25], the Pepper robot was used to answer a set of frequently asked questions of high school students. 18 students interacted with the robot via facilitation by a human presenter who helped with speech recognition. In the remote condition, tele-conferencing was used for the robot. After the interaction, questionnaires based on the Unified Theory of Acceptance and Use of Technology, and the Godspeed Questionnaire were completed. The in-person HRI was considered to have higher perceived sociability and anthropomorphism; however, no significant differences were found in perceived enjoyment, intention to use, trust, intelligence, animacy, and sympathy between the two conditions.

#### 3.1.2. Studies without Differences between Outcomes for in-Person and Remote HRI 

In [53], the Roomba and NAO robots were used as coaches in a visual search task. The task for the adult participants was to identify certain types of targets from the synthetic aperture radar images on a computer, while receiving ambiguous feedback from the robot instructor. For the in-person conditions, the robot was placed next to the computer, while in the remote conditions, real-time robot video was displayed on an additional monitor. The target detection accuracy, inspection time and compliance were measured, but no statistical difference was found between the two conditions. 

In [33], 66 children played a drumming game with the robot Kaspar in three different conditions (in-person, hidden and remote). The in-person conditions consisted of the robot playing drums in front of the participants, and in the remote conditions, Kaspar was projected on a screen in front of the participants. The children’s drum-playing behavior was recorded during the interaction and a Likert-scale questionnaire was used to measure enjoyment, social attraction, involvement, performance, and robot general appearance and intelligence. There were no significant differences reported in game performance (total drumbeats, turn-taking) between the in-person and remote conditions. Furthermore, no specific analysis of the questionnaire results was reported between the in-person and remote conditions. Although, most participants favored the in-person condition, minimal differences were detected in involvement, enjoyment, intelligent, social attraction, and appearance between the in-person and remote conditions. 

In [35], under both in-person and remote conditions, 90 adult participants were guided by the NAO robot to perform physical exercise by following the body movements of the robot. The robot was displayed on a screen in the remote condition. Results from a Likert-scale questionnaire showed no significant differences in users’ ratings on the robot’s intelligence, anthropomorphism, animacy and likability as well as their own anxiety. 

In [36], experiments were conducted with 10 adult participants verbally commanding the Zenbo robot to do tasks such as following, story-telling, weather reporting, etc. In the in-person condition, the robot was placed in front the participant in an outdoor environment, and in the remote condition, it was displayed on a laptop screen. A custom questionnaire was developed based on the Negative Attitude towards Robots Scale, Robotic Social Attributes Scale, the Extended Technology Acceptance Model, the NASA Task Load Index, and the User Experience Questionnaire. Results showed that the participants perceived the in-person HRI and remote HRI similarly in terms of perception and attitudes, however, the remote conditions had a slightly higher workload. 

In [34], the robot Ryan was used to guide users to complete recognition tasks. The tasks involved recognizing robot facial emotions, head orientations and gaze. In the remote condition, the robot’s face was displayed on a screen. No significant difference in the task performance (recognition accuracies) was observed between the two conditions. 

#### 3.1.3. Summary

The aforementioned studies have mainly all used different: (1) robotic platforms, (2) measured outcomes, (3) activities/tasks, (4) participant demographics, and (5) statistical tests. Additionally, the statistical analysis tests were focused on determining if there is a statistically significant difference in a specific measurement between the two conditions, rather than quantitively investigating the effect. Therefore, herein, we provide an across-study comprehensive analysis to investigate differences between remote HRI and in-person HRI conditions and their outcomes. 

### 3.2. Meta-Analysis Procedure

One common challenge of implementing meta-analysis for HRI studies, is that there are usually varying measures used in different studies. In order to address this challenge, we have grouped studies reporting similar HRI outcomes together, similar to the approach presented in [7]. Namely, we group similar *HRI-related outcomes* (considering both human and robot outcomes) into three classes: (1) Positive Experience (PE) of the users, (2) Perceptions and Attitudes (PA) towards the robots, and (3) Efficacy (EF) of the HRI condition. PE represents outcomes related to pleasure and enjoyment experienced during HRI. PA considers outcomes ranging from likeability and trust to human-like features and ease of use. EF considers user outcomes such as task performance, workload, compliance as well as robot outcomes such as competence and social presence. We also consider the overall effect based on all the outcomes reported. Table 2 provides a comprehensive list for each HRI-related outcome class. 

We investigate the aforementioned studies with respect to the three outcome classes identified in Table 2 and the overall combined outcomes based on the means and variances of the measures in each individual study.

In addition to the outcome classes, we consider the following moderating factors:

(1) **Robot Type:** humanoid or non-humanoid;
(2) **Participant Age Group:** children or adults;
(3) **Assistive Activity Type:** (a) information gathering (e.g., engaging in a conversation for the purpose of collecting information from participants), (b) prompting (e.g., providing verbal commands and asking participants to complete certain tasks), (c) facilitating (e.g., coaching and tutoring), (d) recognition (e.g., identifying information in the environment), and (e) answering (e.g., providing answers to the questions asked by the participants);
(4) **User Interaction Modes:** (a) verbal (including spoken speech and speech-to-text input via a keyboard) and/or (b) non-verbal (e.g., object and/or touchscreen manipulation).

We conduct subgroup analysis on these factors to determine if they contribute to differences between the in-person and remote HRI conditions. The recognition activity type [34] and answering activity type [25] are not included in the activity subgroup analysis, as in these cases the subgroup only contains a single study. Subgroup analysis for participants age group is performed between children and adults. Previous meta-analysis for social robots has shown that moderating factors such as application domain, robot design, and characteristics of users directly influence attitudes, acceptance and trust in them [66]. After selecting the aforementioned outcomes and moderators, we computed the effect size for each outcome. We use the small-sample adjusted standardised mean difference Hedges’ *g* [67], and compute an average effect size for each of our outcome classes using an inverse-variance weighting. We set the in-person HRI as positive effect size direction, and remote HRI as the negative effect direction. This means that a positive effect size indicates higher outcomes are observed in the in-person conditions as compared to the remote conditions, on the contrary, higher outcomes in the remote conditions as compared to the in-person conditions result in a negative effect size. The meta-analysis is based on the random-effect model which computes the pooled effect sizes of the outcomes. The assigned weight of each study is determined by its effect size variance [67]. We use inverse-variance weighting, as opposed to weighting by sample size, as a detailed Monte Carlo simulation study has found that inverse-variance weighting leads to smaller mean squared errors and is recommended when using standardized mean difference as the effect size [68]. The analysis was implemented in the R programming language using the {meta} package [69].

## 4. Meta-Analysis Results

We perform meta-analysis and subgroup analysis on the outcomes, when possible, to explore the effects of the remote condition and the in-person condition on outcomes in HRI. The detailed results are presented in this section. 

### 4.1. Overall Effect

**Figure 3:**
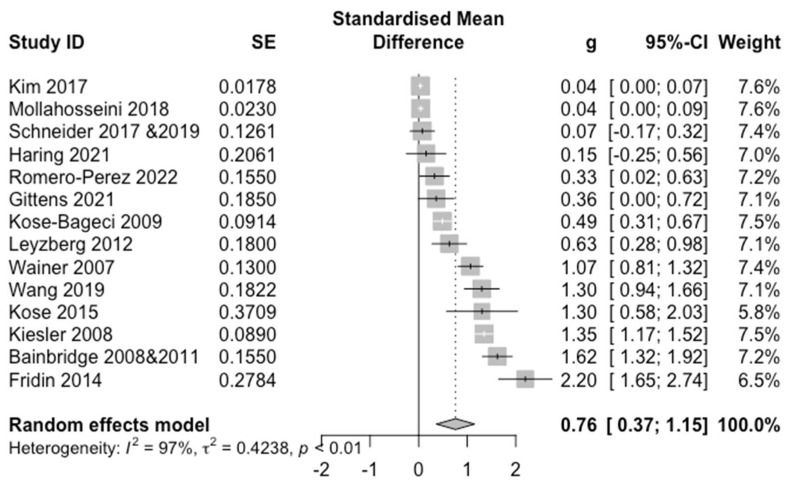
Forest Plot of Overall Effect. For each study and the average effect size, the plot shows standardised mean differences (hedges’ g), standard error (SE), the 95% confidence interval (95% CI), and the weight in the random effect model. Heterogeneity is represented by the between study Higgins & Thompson’s $I^{2}$, heterogeneity variance $\tau^{2}$ and *p*-value.

Figure 3 presents the forest plot of the overall effect of socially assistive robot presence on our HRI outcomes, and depicts Hedges’ *g,* 95% confidence interval (95% CI) and standard error (SE). We also compute Cochran’s *Q*, *p*-value, and Higgins & Thompson’s *I*^2^ for evaluating the between-study heterogeneity. In general, we see a moderate positive overall effect for in-person HRI (*k* = 14, *g* = 0.76, 95% CI = [0.37, 1.15]). A substantial heterogeneity (*Q* = 506.49, *p* < 0.0001, *I*^2^ = 97.4%) indicated that there potentially is diverse variability across our outcomes and with respect to our moderating factors. We then performed subgroup analysis for all the moderators to determine if any of them contribute to the heterogeneity. 

No significant effects from the moderators were found: (1) **humanoid**: *k* = 11, *g* = 0.76, 95% CI = [0.26; 1.25]; **non-humanoid**: *k* = 3, *g* = 0.80, 95% CI = [−0.48; 2.08]; (2) **adults**: *k* = 10, *g* = 0.66, 95% CI = [0.22; 1.10]; **children**: *k* = 4, *g* = 1.04, 95% CI = [−0.32; 2.41]; and (3) **information gathering**: *k* = 3, *g* = 1.01, 95% CI = [−0.36; 2.39]; **prompting**: *k* = 4, *g* = 0.96, 95% CI = [−0.77; 2.70]; and **facilitating**: *k* = 5, *g* = 0.69, 95% CI = [0.16; 1.22]; and (4) **verbal**: *k* = 5, *g* = 0.67, 95% CI = [−0.09; 1.43]; **non-verbal**: *k* = 9, *g* = 0.82, 95% CI = [0.24; 1.40].

Similarly, the *Q*-tests found no significant differences between-subgroups in effect sizes: (1) **robot type**: *Q_M__F_* = 0.01, *p* = 0.91; (2) **participant age group**: *Q_MF_* = 0.67, *p* = 0.41; (3) **assistive activity type**: *Q_MF_* = 0.88, *p* = 0.65; and (4) **user interaction mode**: *Q_MF_* = 0.15, *p* = 0.69. Further analysis for all four moderators found that substantial within-subgroups heterogeneity existed: (1) **humanoid**: *Q* = 300.89, *I*^2^ = 96.7%; **non-humanoid**: *Q* = 30.35, *I*^2^ = 93.4%; (2) **adults**: *Q* = 421.54, *I*^2^ = 97.9%; **children**: *Q* = 41.07, *I*^2^ = 92.7%; (3) **information gathering**: *Q* = 23.57, *I*^2^ = 91.5%; **prompting**: *Q* = 161.93, *I*^2^ = 98.1%; and **facilitating**: *Q* = 22.33, *I*^2^ = 82.1%; and (4) **verbal**: *Q* = 257.15, *I*^2 =^ 98.4%; **non-verbal**: *Q* = 246.44, *I*^2^ = 96.8%. This indicated that the moderators did not have significant influence on the overall effect. Therefore, we then examined PE, PA, and EF separately to investigate more closely the specific effect from each individual moderator. 

### 4.2. Positive Experience 

**Figure 4:**
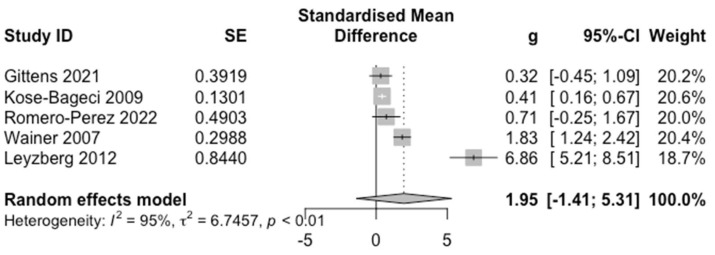
Forest Plot for PE. For each study and the average effect size, the plots show standardised mean differences (hedges’ g), standard error (SE), the 95% confidence interval (95% CI), and the weight in the random effect model. Heterogeneity is represented by the between study Higgins & Thompson’s $I^{2}$, heterogeneity variance $\tau^{2}$ and *p*-value.

Figure 4 presents the forest plot for the PE outcome. A positive effect was observed for in-person HRI (*g* = 1.95). However, the 95% CI had a large range (95% CI = [−1.41, 5.31]), therefore, no specific conclusion can be noted for the effect of robot presence on PE. This large range may be due to the limited number of studies that have focused on PE (*k* = 5). We also see substantial heterogeneity (*Q* = 73.35, *p* < 0.0001, *I*^2^ = 94.5%) showing data variability. 

Since there were only *k* = 5 studies reporting measures related to PE, no subgroup analysis was conducted with respect to assistive activity type, as some subgroups only contain a single study. No significant effect was found from the other moderators: (1) **humanoid**: *k* = 3, *g* = 0.98, 95% CI = [−0.92; 2.88]; **non-humanoid**: *k* = 2, *g* = 3.55, 95% CI = [−38.02; 45.11]; (2) **adults**: *k* = 3, *g* = 2.94, 95% CI = [−5.50; 11.38]; **children**: *k* = 2, *g* = 0.43, 95% CI = [−0.50; 1.36]; and (3) **verbal**: *k* = 2, *g* = 0.47, 95% CI = [−1.97; 2.90]; **non-verbal**: *k* = 3, *g* = 2.96, 95% CI = [−5.38; 11.30].

*Q*-tests found no significant differences between-subgroups in effect sizes for: (1) **robot type**: *Q_MF_* = 0.61, *p* = 0.43; (2) **participant age group**: *Q_MF_* =1.63, *p* = 0.20; and (3) **user interaction mode**: *Q_MF_* = 1.63, *p* = 0.20. Although the *p*-values for participant age group and user interaction mode are not statistically significant, they are relatively small, hence suggesting a potential trend that these subgroups may have an effect on PE. Furthermore, substantial within-subgroups heterogeneity existed for robot type, but not for participant age group and user interaction mode: (1) **humanoid**: *Q* = 18.93, *I*^2^ = 89.4%; **non-humanoid**: *Q* = 49.44, *I*^2^ = 98.0%; (2) **adults**: *Q* = 50.02, *I*^2^ = 96.0%; **children**: *Q* = 0.32, *I*^2^ = 0.0%; and (3) **verbal**: *Q* = 0.39, *I*^2^ = 0.0%, **non-verbal**: *Q* = 31.56, *I*^2^ = 96.8%. This trend also shows that participant age group and user interaction mode could be potential moderators influencing PE outcomes.

### 4.3. Perceptions and Attitudes

**Figure 5:**
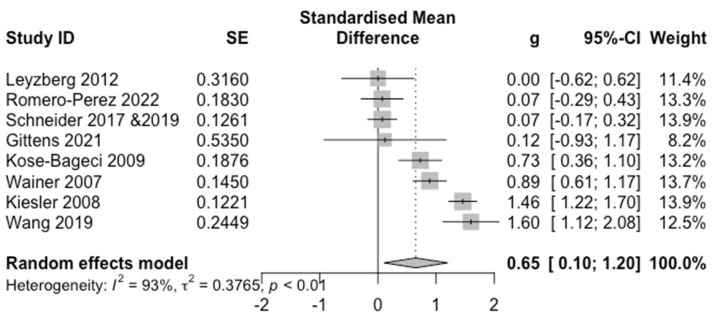
Forest Plot for PA. For each study and the average effect size, the plots show standardised mean differences (hedges’ g), standard error (SE), the 95% confidence interval (95% CI), and the weight in the random effect model. Heterogeneity is represented by the between study Higgins & Thompson’s $I^{2}$, heterogeneity variance $\tau^{2}$ and *p*-value.

Figure 5 presents the forest plot for the PA outcome. A moderate positive effect for in-person HRI (*g* = 0.65) was observed with a small 95% CI ([0.10, 1.20]). We also found substantial heterogeneity (*Q* = 96.04, *p* < 0.0001, *I*^2^ = 92.7%).

For the robot type moderator, no significant effects were found due to the 95% CIs overlapping in range: **humanoid**: *k* = 5, *g* = 0.66, 95% CI = [−0.12; 1.43]; and **non-humanoid**: *k* = 3, *g* = 0.60, 95% CI = [−1.49; 2.68]. A between-subgroup *Q*-test found no significant difference between the humanoid and non-humanoid subgroups on effect sizes (*Q_MF_* = 0.01, *p* = 0.91). A substantial within-subgroup heterogeneity was observed (**humanoid**: *Q* = 45.61, *I*^2^ = 91.2%; and **non-humanoid**: *Q* = 22.97, *I*^2^ = 91.3%). 

As for participant age groups, no significant effect was found: **adults**: *k* = 6, *g* = 0.73, 95% CI = [−0.03, 1.50]; and **children**: *k* = 2, *g* = 0.40, 95% CI = [−3.76; 4.56]. A between-subgroup *Q*-test found no significant difference between these subgroups (*Q_MF_* = 0.57, *p* = 0.45), however, a moderate within-subgroup heterogeneity was observed for children which suggests participant age could potentially influence the PA outcomes: **adults**: *Q* = 81.15, *I*^2^ = 93.8%; and **children**: *Q* = 6.25, *I*^2^ = 54.0%.

For the assistive activity type moderator, no significant difference was also observed for the effect sizes between the subgroups: (1) **information gathering** *(k* =3, *g* = 1.20, 95% CI = [−0.60; 3.01]); and (2) **facilitating**: (*k* = 3, *g* = 0.60, 95% CI = [−0.49; 1.70]). A between-subgroup *Q*-test found no significant difference between-subgroups in effect sizes for the information gathering and facilitating subgroups (*Q_MF_* = 1.47, *p* = 0.226). A low within-subgroup heterogeneity was also observed for **information gathering** (*Q* = 6.52, *I*^2^ = 69.3%) and **facilitating** (*Q* = 6.55, *I*^2^ = 69.5%). The results showed that the effect size difference for the robot type and assistive activity type were not statistically significant. However, based on the $I^{2}$ of each subgroup, assistive activity type contributed to approximately 30% of the variation in the effect size of the studies for the PA outcome. Also considering the *Q*-test result, assistive activity type could potentially influence PA. 

For the user interaction mode moderator, no significant difference was observed for the effect sizes between the subgroups: **verbal**: *k* = 4, *g* = 0.86, 95% CI = [−0.45, 2.18]; and **non-verbal**: *k* = 4, *g* = 0.45, 95% CI = [−0.26; 1.6]). Likewise, no significant results were found by the between-subgroup *Q*-test (*Q_MF_* = 0.77, *p* = 0.38) with substantial within-subgroup heterogeneity observed (**verbal**: *Q* = 47.65, *I*^2^ = 93.7%; **non-verbal**: *Q* = 22.28, *I*^2^ = 86.5%).

### 4.4. Efficacy

**Figure 6:**
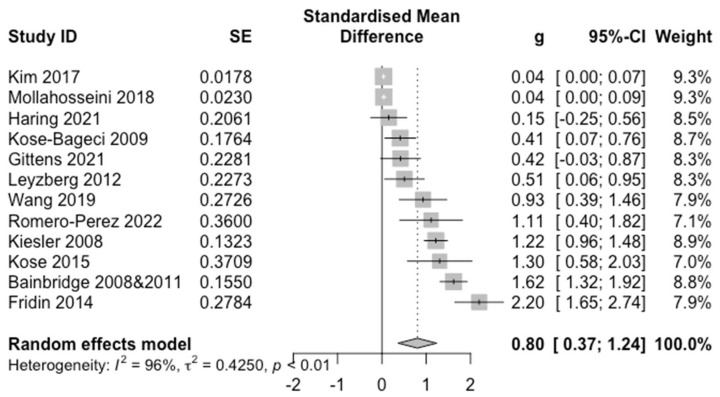
Forest Plot of EF. For each study and the average effect size, the plots show standardised mean differences (hedges’ g), standard error (SE), the 95% confidence interval (95% CI), and the weight in the random effect model. Heterogeneity is represented by the between study Higgins & Thompson’s $I^{2}$, heterogeneity variance $\tau^{2}$ and *p*-value.

Figure 6 shows the forest plot for the EF outcome. A moderate positive effect for in-person HRI (*g* = 0.80) with a 95% CI ([0.37, 1.24]) was found and we also found substantial heterogeneity (*Q* = 277.77, *p* < 0.0001, *I*^2^ = 96%).

Subgroup analysis was performed for all the moderators. No significant effect was determined between the humanoid and non-humanoid robot types: **humanoid**: *k* = 9, *g* = 0.83, 95% CI = [0.24; 1.43]; and **non-humanoid**: *k* = 3, 95% CI = [−0.37; 1.86]. Between-subgroup *Q*-test found no significant difference between-subgroups in effect sizes for the humanoid and non-humanoid subgroups (*Q_MF_* = 0.06, *p* = 0.80). Substantial within-group heterogeneity was observed (**humanoid**: *Q* = 196.60, *I*^2^ = 95.90%; and **non-humanoid**: *Q* = 13.15, *I*^2^ = 84.80%) 

No significant effect was determined between the adult and children subgroups: **adults**: *k* = 8, *g* = 0.61, 95% CI = [0.11; 1.10]; and **children**: *k* = 4, *g* = 1.24, 95% CI = [0.04; 2.44]. Between-subgroups *Q*-test found no significant difference between-subgroups in effect sizes for the participant age groups (*Q_MF_* = 2.14, *p* =0.14), however, the *p*-value was relatively small. Substantial within-subgroups heterogeneity was also observed: **adults**: *Q* = 196.50, *I*^2^ = 96.40%; and **children**: *Q* = 30.27, *I*^2^ = 90.1%. 

There was no effect determined for the assistive activity type due to the overlapping 95% CI ranges: **information gathering**: *k* = 3, *g* = 0.88, 95% CI = [−0.14; 1.90]; **prompting**: *k* = 3, *g* = 1.26, 95% CI = [−1.52; 4.05]; and **facilitating**: *k* = 4, *g* = 0.51, 95% CI = [−0.18; 1.19]. Between-subgroups *Q*-test for activity type found no statistical significance in effect sizes (*Q_MF_* = 2.11, *p* =0.35). We found low within-group heterogeneity in the **information gathering** subgroup (*Q* = 9.23, *I*^2^ = 78.30%) and **facilitating** subgroup (*Q* = 7.47, *I*^2^ = 59.80%), however, substantial heterogeneity was found for the **prompting** subgroup (*Q* = 161.92, *I*^2^ = 98.80). 

For user interaction mode, there was no effect determined due to the overlapping 95% CI ranges: **verbal**: *k* = 5, *g* = 0.71, 95% CI = [0.06; 1.35]; and **non-verbal**: *k* = 7, *g* = 0.86, 95% CI = [0.11; 1.63]. Also, between-subgroups *Q*-test found no statistical significance in effect sizes: *Q_MF_* = 0.17, *p* = 0.68. Substantial within-group heterogeneity was found (**verbal**: *Q* = 99.45, *I*^2^ = 96.0%; **non-verbal**: *Q* = 176.04, *I*^2^ = 96.6%).

Based on this analysis, we found that the four moderators have no statistically significant effect on EF, however, a small *Q*-test *p*-value was found for the participant age group, suggesting a potentially trend that age group may have an effect on efficacy. We also noted that the with-group heterogeneity for the assistive activity type shows a diverse effect due to this moderator, with the facilitating subgroup representing the smallest effect variation. This potentially shows that EF can vary with specific activity type.

### 4.5. Quality of Evidence

Using the GRADE (Grading of Recommendations, Assessment, Development and Evaluation) method [70], the quality of each outcome is also evaluated and presented in Table 3. We note that the PE outcome has a significantly large 95% CI ([−1.41, 5.31]) compared to the overall outcome (95% CI [0.37, 1.15]), PA outcome (95% CI [0.10, 1.20]) and EF outcome (95% CI [0.37, 1.24]), and hence, consider it as a serious limitation in imprecision. Egger’s regression test (*t* = 3.94, *df* = 12, *p* = 0.002) confirms the limitations of small study effects [71], so we consider all the outcomes to have serious limitations in terms of publication bias. Given the above, the quality of evidence is downgraded appropriately.

## 5. Discussion

The key findings of our meta-analysis are that, in general, in-person HRI has a positive effect on the combined outcomes (overall effect) we investigated. Namely, users positively perceive in-person HRI over remote HRI. Furthermore, efficacy was found to be higher for in-person HRI; however, there is no significant evidence to support that positive experience is influenced by the HRI presence type (due to 95% CI range having a negative lower limit). 

Regarding the moderators, robot type, participant age group, assistive activity type and user interaction mode did not have a statistically significant effect on the outcomes as moderators. However, participant age group could potentially influence PE, PA and EF outcomes based on: (1) the small *p*-values obtained for PE and EF, and (2) the low with-in subgroups heterogeneity observed with PE and PA. Previous meta-analysis has also determined that age can be an influential moderator for general robot acceptance [72]. Assistive activity type could potentially influence the PA and EF outcomes due to the low with-in subgroups heterogeneity observed. Interaction mode could also potentially influence PE outcomes based on the small *p*-value and with-in subgroups heterogeneity observed. 

Compared to in-person HRI, lower PA and EF in remote HRI conditions can be contributed by the higher cognitive workload of the users [36]. Previous meta-analysis investigating how people perceive social robots [66] has also found that application or activity has an effect on users’ perceptions and attitudes towards these robots, but no significant effect from the robot’s design and user’s age were found. The reason for the potential difference in age in our analysis may be due to the fact that we were comparing between children and adults age groups, however, in [66], the comparison was between younger and older adults. 

There was no evidence supporting robot type being an influential moderator on any of the outcomes, both given the between-subgroups *Q*-test and with-group heterogeneity. This result is comparable with [73], where a similar effect for robot anthropomorphism was found with both embodied robots and depicted robots. 

### 5.1. Insights

We conclude that participant age group, assistive activity type and user interaction mode have more potential influence on the in-person and remote conditions given low heterogeneity and *p*-values in the subgroup analysis. However, due to the small number of studies, we see large overlapping 95% CI for each subgroup, and therefore, we are not able to draw specific statistical conclusions for each subgroup. A future HRI study could be conducted to directly investigate how participant age groups, specific and varying types of assistive activities and user interaction modes are influenced by in-person and remote robot conditions. 

It is interesting to note that in this meta-analysis, there was no detectable difference between in-person and remote HRI for the PE outcome. In situations where the focus of the HRI is for users to have a positive experience, then remote HRI may be considered a suitable choice, such as for embodied conversational robots [74] and/or companion robots [75]. With the feasibility of remote HRI shown in the studies in the Related Works section of this paper, researchers can explore how remote HRI can be improved in applications that have already shown promise in providing cognitive and social interventions.

Furthermore, more experimental studies between in-person and remote HRI are required to examine other moderating factors, for example, in studies with older adults. As older adults could greatly benefit from interactions with socially assistive robots and have a different set of needs, these needs may be met by both HRI types. For example, older adults have used virtual technologies during the pandemic to meet and chat with family and friends when they were isolated from them. The question of ‘*Could remote robots also help with such activities*?’ is an important one to explore for this specific population. Other demographic factors such as sex, gender, and culture should also be investigated. 

An advantage of remote HRI is its potential to scale up interactions and enable several users in their own home environments to interact with a single robot remotely, whether at the same time or consecutively and as remote groups. The studies presented herein have all used either a projector [33,48] or a monitor [34,35,36,48,49,50,51,54,55] as visualization tools to present the remote robot. With the popularity of virtual reality (VR) and its potential use in HRI [76], the possibility of integrating VR for remote HRI systems could also be explored to emerge the user in the same environment as robots [77]. 

### 5.2. Considerations and Limitations

It is important to note that only a small group of studies to date has compared in-person HRI and remote HRI, with a handful of outcome measures. As a result, we were only able to investigate three outcomes (PE, PA, and EF) and four moderators (robot type, participant age group, assistive activity type and user interaction mode). For each study, similar outcomes were grouped together to determine the weighted average effects and the overall effect, assuming individual outcomes were independent. This could lead to the risk of underestimating the overall variance of effect sizes [78]. However, since none of the studies included in our meta-analysis reported correlations between the outcomes, we believe this risk is minimal. Furthermore, the size of studies included in our analysis is comparable to other meta-analyses conducted for HRI on trust [3,79], robot personality and human acceptance [80], and questionnaire usage [81]. We observed a substantial heterogeneity in each of our subgroups, indicating that the moderators used may not be the only moderators to consider for in-person HRI and remote HRI. Various other moderating factors (not reported in the studies considered herein) may have influenced the PE, PA and EF as well. 

## 6. Conclusions

In this paper, we present a meta-analysis to investigate the influence of in-person and remote HRI with socially assistive robots on user positive experience, perceptions and attitudes, and efficacy. Our results confirmed the tendency toward in-person HRI over remote HRI in terms of the overall effect of the combined outcomes, as well as the outcomes of perceptions and attitudes, and efficacy; however, not for the positive experience outcome showing the potential for interactions with remote robot presence. Our findings also suggest that age group is most related to positive experience, users’ perceptions, and attitudes, and efficacy; assistive activity type is most related to users’ perceptions and attitudes, and efficacy; and user interaction mode is most related to positive experience. 

Future research should focus on conducting more in-person HRI and remote HRI studies considering varying tasks, demographics, and robot types in order to obtain a deeper understanding of when, and for what, assistive tasks these two HRI conditions should be used and would be effective for. In particular, the consideration of older adult participants for in-person and remote HRI should be investigated, as they are an important user group who can directly benefit from assistance with rehabilitation and daily activity tasks. The use of remote HRI to provide assistance with daily activities and interventions is a promising emerging field for use in promoting health and well-being and should be investigated further.

## Figures and Tables

**Table 1:** Summary of the Remote and In-person HRI Studies.

Study ID	Robot Type	# of Participants	Participant Age Group	Activity	User Interaction Modes
Wainer 2007 [49]	Non-humanoid 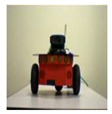 ActivMedia Pioneer 2 DX, courtesy of Maja J. Matarić [49]	21	Adults	Facilitating	Non-verbal
Kiesler 2008 [48]	Humanoid 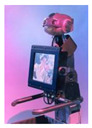 Pearl robot, courtesy of National Science Foundation	113	Adults	Information gathering	Verbal
Bainbridge 2008 & 2011 [54,63]	Humanoid 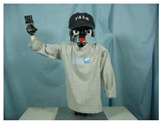 Nico robot, courtesy of Brian Scassellati [54]	65	Adults	Prompting	Non-verbal
Leyzberg 2012 [50]	Non-humanoid 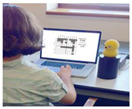 Keepon, courtesy of Brian Scassellati [50]	100	Adults	Facilitating	Non-verbal
Kose-Bageci 2009 [33]	Humanoid 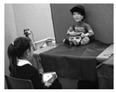 Kaspar robot, courtesy of Kose-Bageci et al. [33]	100	Children	Facilitating	Non-verbal
Fridin 2014 [51]	Humanoid 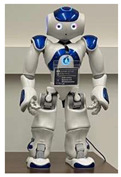 NAO robot, courtesy of ASBLab	9	Children	Prompting	Non-verbal
Schneider 2017 & 2019 [35,64]	Humanoid 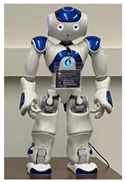 NAO robot, courtesy of ASBLab	90	Adults	Prompting	Non-verbal
Kim 2017 [46]	Humanoid robot 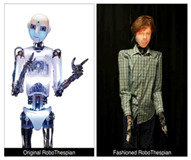 RoboThespian robot, courtesy of Kim et al. [46]	7685	Adults	Prompting	Verbal
Gittens 2021 [36]	Non-humanoid 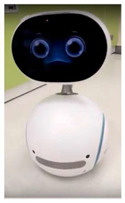 Zenbo robot [65], under a Creative Commons Attribution 4.0 International License	10	Adults	Information gathering	Verbal
Kose 2015 [52]	Humanoid 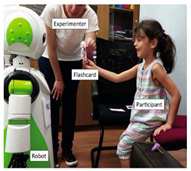 Robovie R3 robot, courtesy of Kose et al. [52]	31	Children	Facilitating	Non-verbal
Haring 2021 [53]	Humanoid 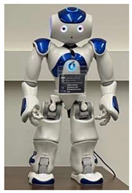 Nao robot, courtesy of ASBLab	60	Adults	Facilitating	Non-verbal
Wang 2019 [55]	Humanoid 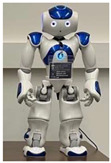 Nao robot, courtesy of ASBLab	60	Adults	Information gathering	Verbal
Mollahosseini 2018 [34]	Humanoid 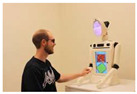 Ryan robot, courtesy of Mollahosseini et al. [34]	17	Adults	Recognition	Non-verbal
Romero-Perez 2022 [25]	Humanoid 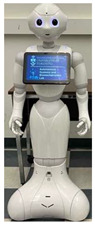 Pepper robot, Nao robot, courtesy of ASBLab	18	Children	Answering	Verbal

**Table 2:** Summary of the Remote and In-person HRI Studies.

Outcome Classes	Related Research Question
**Positive experience (PE):** user experience during interactions including pleasure and enjoyment.	Do humans have a better experience with in-person HRI or remote HRI?
**Perceptions and Attitudes towards the robots (PA):** likeability and intelligence, helpfulness, human-likeness, trust, acceptance, respect and ease of use.	Do humans perceive robots differently under the in-person and remote conditions?
**Efficacy (EF):** user performance measures including task performance, activity level, workload, compliance, ability and robot influence. Robot performance measures including social presence and competence.	Does HRI performance differ under in-person and remote conditions?

**Table 3:** Quality of evidence based on the GRADE method.

Outcome	Risk of Bias	Inconsistency	Indirectness	Imprecision	Publication Bias	Quality of Evidence
Overall	Not serious	Not serious	Not serious	Not serious	Serious	Moderate
PE	Not serious	Not serious	Not serious	Serious	Serious	Low
PA	Not serious	Not serious	Not serious	Not serious	Serious	Moderate
EF	Not serious	Not serious	Not serious	Not serious	Serious	Moderate

## Data Availability

Not applicable.
